# How do climate-related uncertainties influence 2 and 1.5 °C pathways?

**DOI:** 10.1007/s11625-017-0525-2

**Published:** 2018-01-09

**Authors:** Xuanming Su, Hideo Shiogama, Katsumasa Tanaka, Shinichiro Fujimori, Tomoko Hasegawa, Yasuaki Hijioka, Kiyoshi Takahashi, Jingyu Liu

**Affiliations:** 1 Center for Social and Environmental Systems Research, National Institute for Environmental Studies (NIES), Tsukuba, Japan; 2 Center for Global Environmental Research, National Institute for Environmental Studies (NIES), Tsukuba, Japan; 3 Energy Program, International Institute for Applied Systems Analysis (IIASA), 2361 Laxenburg, Austria; 4 Ecosystem Services and Management Program, International Institute for Applied Systems Analysis (IIASA), 2361 Laxenburg, Austria

**Keywords:** Climate change, Climate-related uncertainties, Socioeconomic scenarios, Carbon prices, Mitigation costs, Adaptation costs

## Abstract

We investigate how uncertainties in key parameters in the carbon cycle and climate system propagate to the costs of climate change mitigation and adaptation needed to achieve the 2 and 1.5 °C targets by 2100 using a stochastic version of the simple climate model for optimization (SCM4OPT), an integrated assessment model. For the 2 °C target, we find a difference in 2100 CO_2_ emission levels of 20.5 GtCO_2_ (− 1.2 GtCO_2_ to 19.4 GtCO_2_), whereas this difference is 12.0 GtCO_2_ (− 6.9 GtCO_2_ to 5.1 GtCO_2_) for the 1.5 °C target (17–83% range). Total radiative forcing in 2100 is estimated to be 3.3 (2.7–3.9) Wm^−2^ for the 2 °C case and 2.5 (2.0–3.0) Wm^−2^ for the 1.5 °C case. Carbon prices in 2100 are 482 (181–732) USD(2005)/tCO_2_ and 713 (498–1014) USD(2005)/tCO_2_ for the 2 and 1.5 °C targets, respectively. We estimate GDP losses in 2100 that correspond to 1.9 (1.2–2.5)% of total gross output for the 2 °C target and 2.0 (1.5–2.7)% for the 1.5 °C target.

**Electronic supplementary material:**

The online version of this article (10.1007/s11625-017-0525-2) contains supplementary material, which is available to authorized users.

## Introduction

The Paris Agreement aims to hold the global temperature rise in this century to well below 2 °C relative to pre-industrial levels and to pursue efforts to limit the temperature increase to 1.5 °C. Integrated assessment models (IAMs), which account for the interactions among the socioeconomic and physical aspects of climate change, are used to explore emission pathways for achieving climate change targets. Considerable uncertainties surround IAMs regarding both socioeconomics and climate science (Rotmans and van Asselt 2001; van Asselt and Rotmans 2002; Heal and Millner 2014; Gillingham et al. 2015). In terms of these socioeconomic aspects, recent IAM studies have investigated uncertainties stemming from socioeconomic impacts, technological change, and discount rates (Rotmans and van Asselt 2001; van Asselt and Rotmans 2002; van den Bergh and Botzen 2014, 2015; Gillingham et al. 2015; Metcalf and Stock 2015; Weyant 2017). However, the focus is usually placed on limited sources of uncertainty in the physical climate aspects. For instance, three IAMs that are widely used to evaluate the social cost of carbon, DICE (Nordhaus and Sztorc 2013; Nordhaus 2013, 2014), FUND (Anthoff et al. 2011), and PAGE (Hope 2011, 2013) address primarily the uncertainty in (equilibrium) climate sensitivity. Other sources of uncertainty, including those arising from the carbon cycle and the effects of aerosols, are not considered explicitly in these IAMs.

In this study, we employ the simple climate model for optimization (SCM4OPT) (Fujimori et al. 2016, Su et al. 2017), an IAM that considers a full suite of greenhouse gases, pollutants, and aerosols. Here, we probe the uncertainties arising from various physical and biogeochemical processes, using sets of parameter estimates obtained from model emulations. As a note of caution, this analysis does not address potentially larger uncertainties that can be seen from observational constraints based on inversion approaches (Tanaka et al. 2009; Bodman et al. 2013). We consider only uncertainties that result from physical climate processes and hold the socioeconomic parameters constant. The objectives of this study are (1) to evaluate emission pathways that limit the temperature increase to below 2 °C or 1.5 °C after 2100 while considering several sources of climate-related uncertainties and (2) to explore how such climate-related uncertainties are translated into uncertainties in socioeconomic quantities, such as carbon prices, mitigation costs, adaptation costs, and GDP losses.

## Methodology

### Model description

**Fig. 1:**
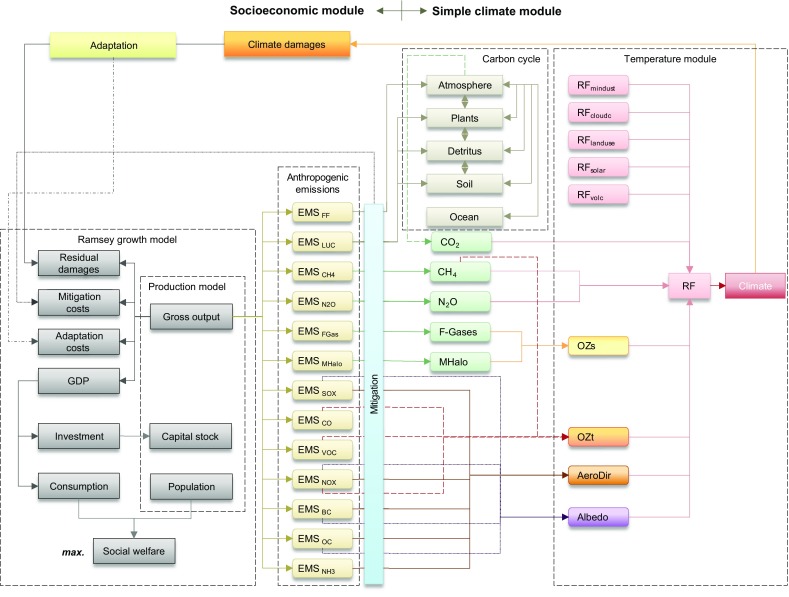
SCM4OPT model structure. The abbreviations shown in this figure are as follows. *AeroDir* direct forcing effects from aerosols, *cloudc* cloud cover, *EMS* emissions, *FF* fossil fuels, *GDP* gross domestic product, *LUC* land-use change, *max* maximize, *MHalo* halogenated gases regulated under the Montreal Protocol, *mindust* mineral dust, *OZs* stratospheric ozone, *OZt* tropospheric ozone, *RF* radiative forcing, *volc* volcanic

SCM4OPT consists of a socioeconomic module and a simple climate module (Fig. 1). The socioeconomic module is calibrated to represent the Shared Socioeconomic Pathways 2 (SSP2) scenario (Fujimori et al. 2017, Su et al. 2017). We select the Asia–Pacific Integrated Assessment/Computable General Equilibrium (AIM/CGE) (Fujimori et al. 2014a, b, 2017), which is one of the IAMs that can implement the SSPs, for use in our working team. We use this model to generate the SSP2 assumptions based on the SSP narratives to ensure model consistency with our earlier study (Su et al. 2017), although AIM/CGE was used to produce the marker scenario of SSP3. As in the DICE-2013R model (Nordhaus and Sztorc 2013; Nordhaus 2013), the global gross production output is defined using a constant-returns-to-scale Cobb–Douglas production function (Cobb and Douglas 1928; Douglas 1976), considering capital, population, and Hicks-neutral technological change (Hicks 1966). The gross output is, therefore, distributed into GDP, adaptation costs, mitigation costs, and residual damages that result from feedbacks on climate change. We maximize the total social welfare, a discounted sum of the utility of per capita consumption, weighted by population. The approach used here is based on the Ramsey economic growth model (Ramsey 1928). We endogenously account for the emission abatements of not only industrial CO_2,_ but also land-use CO_2_, CH_4_, N_2_O, halogenated gases, CO, volatile organic compounds (VOCs), SO_*x*_, NO_*x*_, black carbon (BC), and organic carbon (OC) through the use of individual marginal abatement cost (MAC) curves. The abatement levels of non-CO_2_ components are defined as a power function of control levels of industrial CO_2_ emissions that is based on a sensitivity analysis of the SSP2 scenario (Su et al. 2017). Therefore, the abatement costs and the carbon prices are attributed only to reductions in industrial CO_2_ emissions in this study. Negative CO_2_ emissions from industrial sources can be realized through carbon capture and storage (CCS). The land-use emissions considered here include CO_2_ emissions from land use and land-use changes, including pasture conversion, deforestation, afforestation, reforestation, and soil management (Fujimori et al. 2017, Su et al. 2017). The net land-use emissions are determined by the base case emissions subtracting the abated amount that relates to the reduction level of industrial CO_2_ emissions [see Eqs. (4–5) in the Supporting Information of Su et al. (2017)]. The resulting net emissions are fed into the simple climate module to calculate the radiative forcing and the global mean temperature (GMT) change relative to pre-industrial levels. The simple climate module is largely consistent with MAGICC 6.0 (Meinshausen et al. 2011a, b), except for a few processes that are simplified in SCM4OPT. For instance, we use a two-box temperature module to estimate the GMT, instead of the upwelling-diffusion climate model used in MAGICC 6.0. Therefore, the ocean heat uptake is not explicitly considered in this study. We estimate the climate change damages using a damage function related to the GMT increase and consider both mitigation and adaptation options:

1 $${\Delta _{{\text{gross}}}}(t)={Y_{{\text{gross}}}}(t)({a_1}T(t)+{a_2}T{(t)^{{a_3}}}),$$

where $\varDelta$_gross_(*t*) is the gross damage, *Y*_gross_(*t*) represents the gross output, and $T\left(t\right)$ denotes the GMT. The $t$ stands for annual time. The parameters ${a}_{1}$, ${a}_{2,}$ and ${a}_{3}$ are estimated to be 0.0005, 0.0025, and 2.2523, respectively (Su et al. 2017).

### Uncertainties

Our analysis accounts for the uncertainties in a total of 35 key physical and biological parameters that represent the inter-model differences among 10 C^4^MIP carbon cycle models and 20 CMIP3 AOGCMs (Tables S1–S4). Broadly, our approach is as follows. For processes that are consistent with MAGICC6, we adopt the values of the associated parameters estimated for MAGICC6 to emulate the inter-model comparison results. On the other hand, for processes that are simplified from MAGICC6, we tune the associated parameters to provide a best fit to the emulation results from MAGICC6.

More specifically, 30 parameters (nos. 1–30 in Tables S1–S3) are adjusted to mimic the output from MAGICC 6.0 for each C^4^MIP carbon cycle model, namely, BERN, CCSM1, CLIMBER, FRCGC, HADLEY, IPSL, LLNL, MPI, UMD2, and UVIC. Two parameters related to CO_2_ fertilization (nos. 23–24 in Table S2) and six parameters used to calculate human-induced regrowth of the land biosphere (nos. 25–30 in Table S3) are tuned to provide a best fit to each of the emulated atmospheric CO_2_ concentration pathways extending to 2100 from MAGICC 6.0. All of the other carbon cycle parameters are assumed to be the same as those used in MAGICC 6.0 (nos. 1–22 in Table S1). The calibration results for atmospheric CO_2_ concentrations are shown in Figure S1. The mean temporal evolution of the CO_2_ concentrations is similar between MAGICC6.0 and SCM4OPT, but the uncertainty ranges are relatively large in the latter, due to the simplified treatments of CO_2_ fertilization effects and land-use emissions due to regrowth used in that model. For instance, the emulation results of CO_2_ concentration in 2100 generated by SCM4OPT are $441_{{ - 35}}^{{+47}}$, $565_{{ - 64}}^{{+80}}$, $700_{{ - 93}}^{{+98}}$, and $961_{{ - 145}}^{{+129}}$ parts per million (ppm) for the Representative Concentration Pathway (RCP) scenarios RCP3PD, RCP4.5, RCP6, and RCP8.5 (Vuuren et al. 2011; Thomson et al. 2011; Masui et al. 2011; Riahi et al. 2011), respectively (the subscript and superscript numbers indicate the 17–83% range). The corresponding estimates produced by MAGICC 6.0 are $435_{{ - 29}}^{{+16}}$, $559_{{ - 47}}^{{+34}}$, $696_{{ - 75}}^{{+52}}$, and $969_{{ - 124}}^{{+80}}$ ppm.

Furthermore, we use five parameters in the two-box temperature module to capture the behavior of several CMIP3 AOGCMs (i.e., CCCMA_CGCM3_1_T47, CNRM_CM3, CSIRO_MK3_0, GFDL_CM2_0, GFDL_CM2_1, GISS_MODEL_E_H, GISS_MODEL_E_R, IAP_FGOALS1_0_G, INMCM3_0, IPSL_CM4, MEDIUM_CMIP3_ECS3, MIROC3_2_HIRES, MIROC3_2_MEDRES, MIUB_ECHO_G, MPI_ECHAM5, MRI_CGCM2_3_2A, NCAR_CCSM3_0, NCAR_PCM1, UKMO_HADCM3, and UKMO_HADGEM1). We adopt the estimates of climate sensitivity and the forcing associated with CO_2_ doubling from the emulation results obtained using MAGICC6 (nos. 31–32 in Table S4) and tune the other three parameters against the GMT projections to 2100 (nos. 33–35 in Table S4). The calibrated temperature results shown in Figure S2 indicate that the results obtained using MAGICC6.0 and SCM4OPT are similar; however, the uncertainty ranges obtained using the latter model are slightly larger. These larger uncertainty ranges are partly propagated from the carbon cycle and result partly from the climate system.

Compared to the DICE model or other optimization IAMs, such as FUND and PAGE, SCM4OPT explicitly considers both mitigation and adaptation climate options with active feedbacks from climate change using the state-of-the-art scenario assumptions and covers a full suite of anthropogenic emissions that are of great importance for assessing low climate targets with stabilization (Su et al. 2017). However, the uncertainty ranges of CO_2_ concentrations are larger than the results obtained using MAGICC 6.0, because the treatments of some processes are simplified to enable the complex evolution of the carbon cycle to be incorporated into the optimization procedure used in the IAM, and simplified processes also exist in the climate module. We calibrate the temperature output to be consistent with the results obtained using MAGICC 6.0; thus, the climate change-related uncertainties can be minimized.

### Experimental setup

This study uses the SSP2 as a socioeconomic projection for the future. The SSP2 describes a historical development pattern of moderate challenges within both mitigation and adaptation (Fricko et al. 2017). We explore three cases. (1) in BaseC, no climate policy is implemented in the future; (2) in 20DEG, the temperature increase is kept below 2 °C; and (3) in 15DEG, the temperature increase is kept below 1.5 °C. In this analysis, we assume that the temperature targets are met by the end of this century.

We evaluate 200 parameter combinations for each case and calculate the quantiles corresponding to the given probabilities for individual years. We follow the intervals used in the IPCC definition of likelihood (IPCC 2007). Specifically, the (1) median indicates the median of the probability distribution; (2) likely indicates a 66% probability or the percentile range of 17–83%; and (3) extremely likely indicates a 95% probability or the percentile range of 2.5–97.5%. Throughout this paper, the ranges indicated in the text are likely ranges, unless noted otherwise.

Our uncertainty analysis is conducted based on C^4^MIP and CMIP3 to allow comparison with the MAGICC 6.0 results (Meinshausen et al. 2011a, b). A newer model inter-comparison, CMIP5, was released in 2013 (Taylor et al. 2011) for the Fifth Assessment Report (AR5) of the IPCC. It is reported that the mean values and ranges of the GMT changes simulated by the CMIP5 and CMIP3 models are generally consistent [Box TS.6 of IPCC (2013)]. Hence, using the CMIP5 models would not substantially affect our conclusions.

## Results and discussion

### Emission reductions for 2 and 1.5 °C

**Fig. 2:**
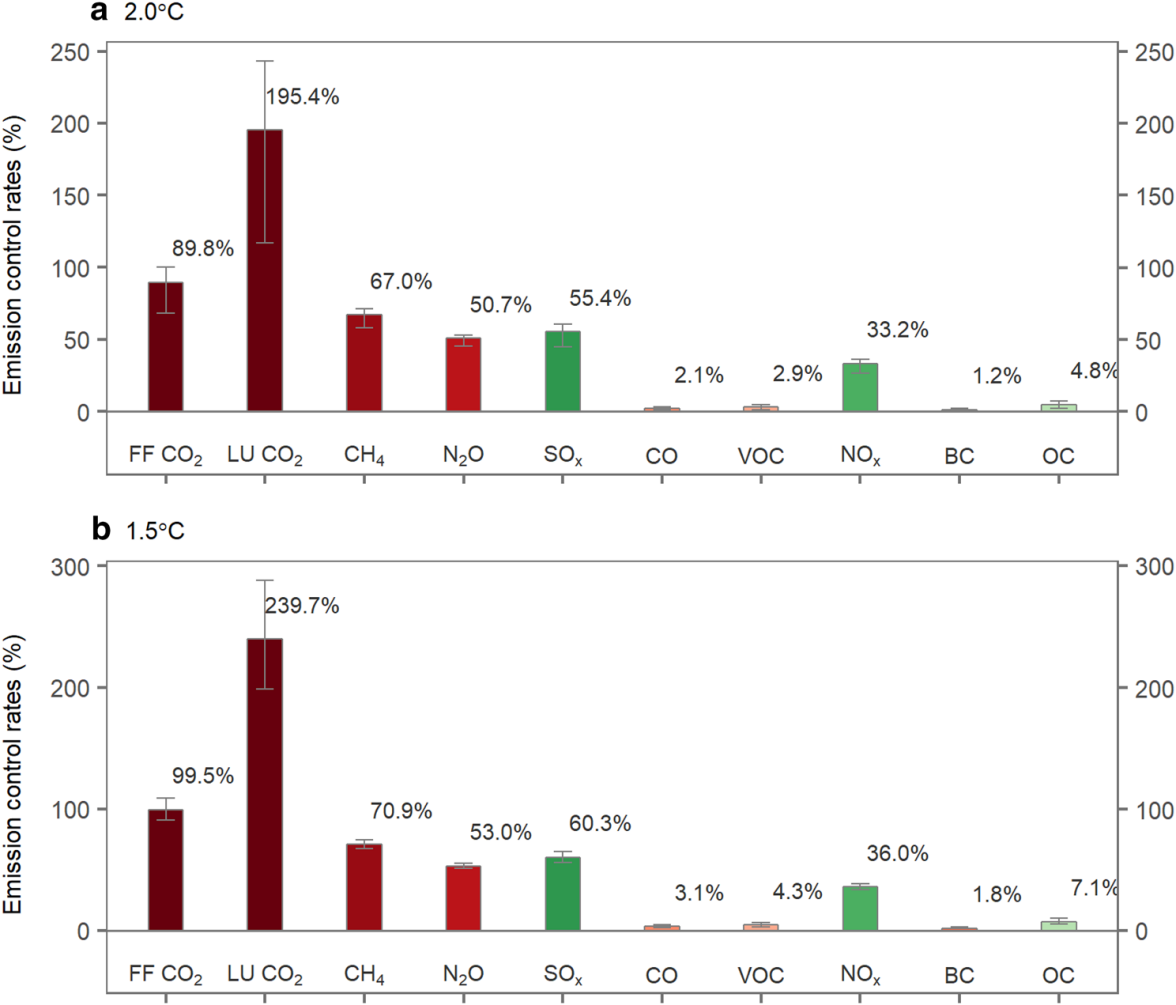
Control rates of anthropogenic emissions in 2100. **a** 2 °C case; **b** 1.5 °C case. The CO_2_ emissions include emissions from fossil fuel combustion and industrial processes (FF CO_2_) and land use (LU CO_2_). The bars indicate the median estimates; the lower and upper bounds of the error bars represent the 17th and 83rd percentiles

We calculate the control rates of individual emissions from each run using the BaseC scenario as a reference and determine the uncertainty ranges for emission reductions with the methods described above. As shown in Fig. 2, the climate-related uncertainties result in wide ranges of reductions in GHGs and aerosols. First, for the 2 °C case, the industrial CO_2_ control rate in 2100 is $90_{{ - 21}}^{{+10}}$%, which corresponds to $97_{{ - 0}}^{{+1}}$% of the total CO_2_ reductions, and that for the land-use CO_2_ emissions is $195_{{ - 78}}^{{+48}}$%, which accounts for $3_{{+0}}^{{ - 1}}$% of the total CO_2_ reductions. For the 1.5 °C case, the reduction rate reaches up to $99_{{ - 9}}^{{+9}}$% for the industrial CO_2_ emissions (accounting for $97_{{ - 0}}^{{+0}}$% of the total CO_2_ reductions) and $240_{{ - 41}}^{{+49}}$% for the land-use CO_2_ emissions (accounting for $3_{{ - 0}}^{{+0}}$% of the total CO_2_ reductions). Control rates larger than 100% indicate net negative emissions. Thus, within the likely range, net negative industrial CO_2_ emissions are not indicated for the 2 °C case by the end of this century. In contrast, there is an approximately 50% chance that the net CO_2_ emissions from industrial sources become negative in the 1.5 °C case. The upper bound (19.4 GtCO_2_) and the lower bound (− 1.2 GtCO_2_) yield a difference of 20.5 GtCO_2_ for the total CO_2_ emissions in 2100 for the 2 °C target. The corresponding difference for the 1.5 °C target is narrower (i.e., 12.0 GtCO_2_; 5.1 GtCO_2;_ and − 6.9 GtCO_2_ are the upper and lower bounds). Second, the control rates of CH_4_ and N_2_O are $67_{{ - 9}}^{{+4}}$ and $51_{{ - 5}}^{{+2}}$% for 2 °C and $71_{{ - 4}}^{{+4}}$ and $53_{{ - 2}}^{{+2}}$% for 1.5 °C, respectively, because these reductions are assumed to occur mainly from industrial sources, and these abatement potentials are relatively limited under the SSP2 assumptions, compared to reductions in CO_2_ emissions. Third, comparatively smaller fractions of SO_x_, NO_x_, and other aerosols and pollutants are removed because of their small baseline emissions, which are due to the intensive air pollution controls implicit in SSP2 (Fujimori et al. 2017; Rao et al. 2017, Su et al. 2017).

The likely cumulative CO_2_ emissions during 2011–2100 are $1665_{{ - 752}}^{{+639}}$ GtCO_2_ for the 2 °C target and $802_{{ - 599}}^{{+752}}$ GtCO_2_ for the 1.5 °C target. These results are larger than previous estimates summarized in the AR5 Synthesis Report (IPCC 2014b; see Table 2.2); the carbon budgets (2011–2100) to achieve 2 and 1.5 °C stabilization with a medium likelihood are 1150–1400 GtCO_2_ and 550–600 GtCO_2_, respectively. We speculate that the differences come from the fact that our results achieve the temperature targets in 2100 after an overshoot; in contrast, many of the IPCC AR5 scenarios achieve the targets before 2100 and reduce the temperatures further, leading to smaller carbon budgets.

### Radiative forcing

**Fig. 3:**
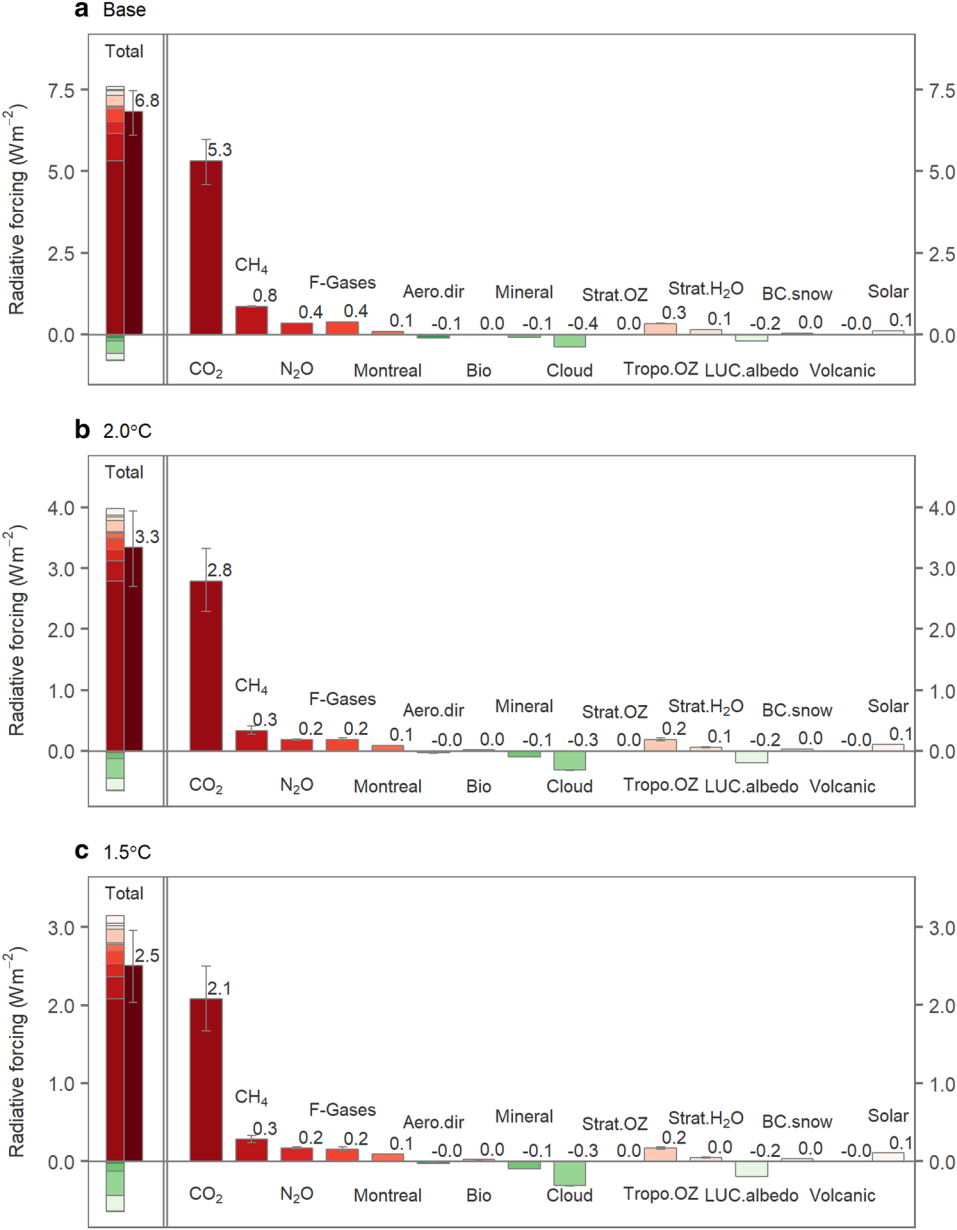
Radiative forcing in 2100. **a** Base case; **b** 2 °C case; **c** 1.5 °C case. Total radiative forcing includes anthropogenic forcings (from GHGs and the direct effects of aerosols, including SO_*x*_, NO_*x*_, OC, BC, biomass and mineral dust, cloud cover and albedo, stratospheric ozone, tropospheric ozone, and stratospheric water vapor from CH_4_ oxidation) and natural forcings [such as volcanic and solar irradiance changes, which are assumed to be 0.0 and 0.1 Wm^−2^ after 2005, respectively (Meinshausen et al. 2011a)]. In the left-hand frame, both the positive and negative forcings are explicitly shown in the bar to the left, and the net forcing obtained by summing all of the forcings is shown by the bar to the right. The right-hand frame shows individual forcing components. The bars indicate the median estimates; the lower and upper bounds of the error bars indicate the 17th and 83rd percentiles

Radiative forcing is used to distinguish the causes of perturbations in the climate system. The uncertainty range for each radiative forcing component is shown in Fig. 3. Here, the radiative forcing for the base case is $6.8_{{ - 0.7}}^{{+0.7}}$ Wm^−2^ in 2100, leading to a GMT increase of $3.8_{{ - 0.6}}^{{+0.7}}$ °C. This result is consistent with the marker scenario of SSP2 (Fricko et al. 2017). For the 2 °C case, the total forcing in 2100 is ${3.3}_{-0.6}^{+0.6}$ Wm^−2^, and this quantity is ${2.5}_{-0.5}^{+0.4}$ Wm^−2^ for the 1.5 °C case. As for the individual forcing agents, the largest forcing uncertainty comes from CO_2_, for which the estimate is ${2.8}_{-0.5}^{+0.5}$ Wm^−2^ in 2100 for the 2 °C case and ${2.1}_{-0.4}^{+0.4}$ Wm^−2^ for the 1.5 °C case. The magnitudes and uncertainty ranges of the other radiative forcing terms are relatively small. Compared to the AR5 transformation pathways, the radiative forcing level for the 2 °C case falls within the category with a range of 430–480 ppm CO_2_-eq with an overshoot of < 0.4 Wm^−2^, whereas the 1.5 °C case is located within the category with a range of 480–530 CO_2_-eq ppm with an overshoot of < 0.4 Wm^−2^ [see Tables 6.2 and 6.3 in IPCC (2014a)].

### Economic costs

**Fig. 4:**
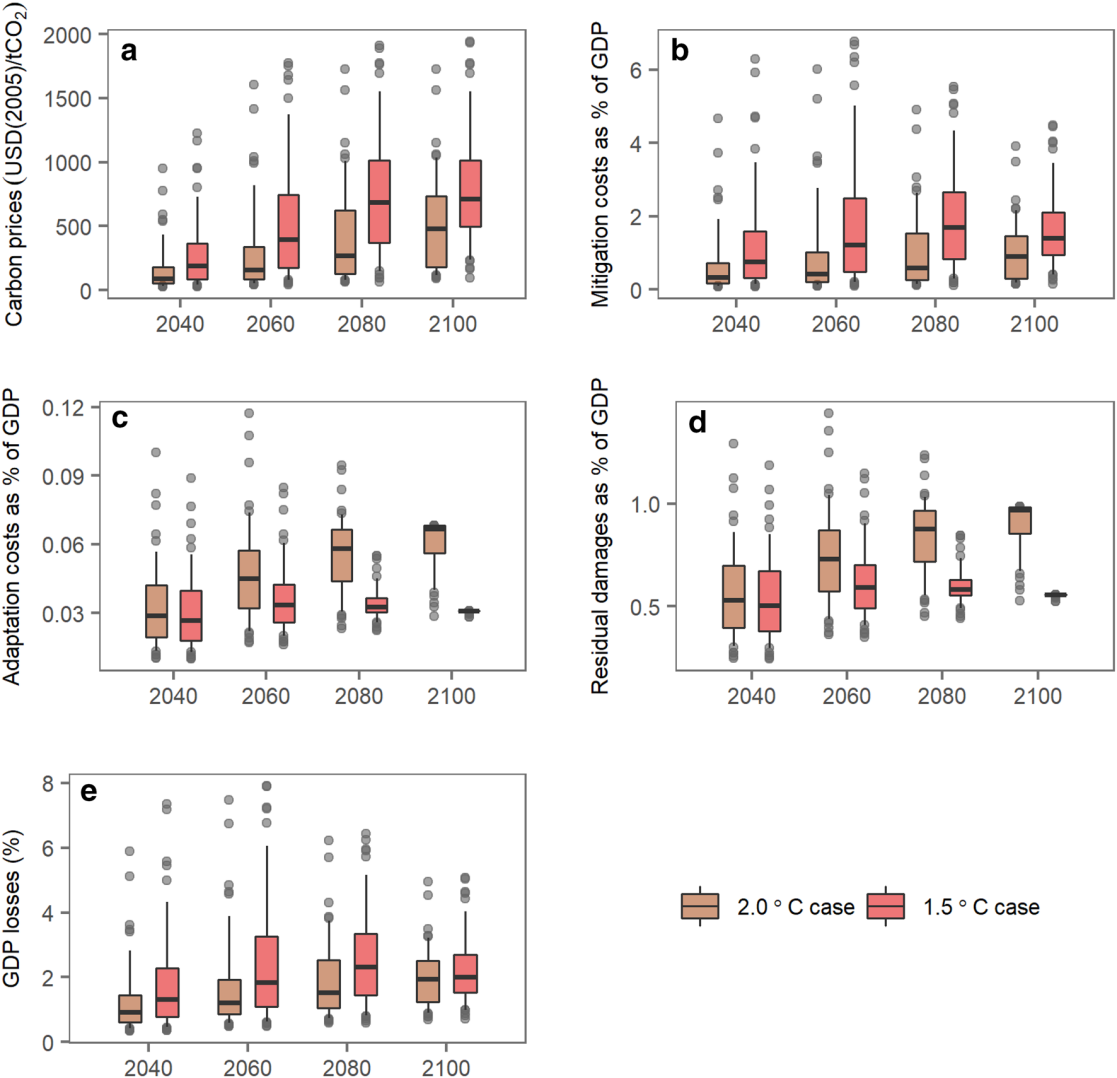
Economic costs of climate change. **a** Carbon prices. **b** Mitigation costs. **c** Adaptation costs. **d** Residual damages. **e** GDP losses. The adaptation assumptions are based on crude estimates obtained using AD-DICE (de Bruin et al. 2009; de Bruin and Dellink 2011); we re-estimated the parameters using DICE-2013R (Su et al. 2017). The middle horizontal lines are the medians; the ranges between the lower and upper hinges show the likely probabilities (i.e., the 17th and 83rd percentiles); the ranges between the lower and upper whiskers indicate the extremely likely probabilities (i.e., the 2.5th and 97.5th percentiles); and the points represent outliers beyond the likely ranges

The carbon prices increase from ${90}_{-38}^{+91}$ USD(2005)/tCO_2_ in 2040 to ${482}_{-301}^{+250}$ USD(2005)/tCO_2_ in 2100 for the 2 °C case (Fig. 4a). The median carbon prices for the 1.5 °C case are 1.5 times greater than those for the 2 °C case: ${189}_{-102}^{+178}$ USD(2005)/tCO_2_ in 2040 and ${713}_{-215}^{+301}$ USD(2005)/tCO_2_ in 2100. The carbon prices corresponding to the upper ends of the uncertainty ranges are 129–552 USD(2005)/tCO_2_ higher than those corresponding to the lower ends for the 2 °C case in the results and 280–516 USD(2005)/tCO_2_ for the 1.5 °C case. The average annual mitigation costs in the 1.5 °C case are roughly twice those obtained for the 2 °C case in the same period. Differences can also be identified in the second half of this century, due to the climate-related uncertainties. These differences correspond to 0.5–1.3% of total gross output for the 2 °C case and 1.2–2.0% of total gross output for the 1.5 °C case (Fig. 4b). However, the adaptation costs in 2100 for the 1.5 °C case (0.03% of total gross output) are approximately half those obtained for the 2 °C case (0.05% of total gross output), because stringent climate targets require less adaptation to avoid climate damages (Fig. 4c). The total climate costs resulting from mitigation, adaptation and residual damages (Fig. 4d), which can be expressed as GDP losses, are shown in Fig. 4e. The GDP losses are % and ${1.3}_{-0.5}^{+1.0}$% in 2040 for the 2 °C and 1.5 °C cases and increase to ${1.9}_{-0.7}^{+0.6}$ and ${2.0}_{-0.5}^{+0.7}$% in 2100, respectively.

The results indicate that climate-related uncertainties have large impacts on economic assessments of climate change. First, the upper-end carbon prices are approximately quadruple those at the lower ends for both the 2 and 1.5 °C climate targets in the second half of this century. Second, moderate GDP losses occur as a result of achieving the climate targets. However, the upper-end GDP losses are also double those of the lower end for the 2 °C target, whereas the corresponding factor is 3 for the 1.5 °C target in the same period. Third, both the carbon prices and the GDP losses are reduced, and their ranges become relatively small in 2100 compared to earlier periods, because we assume adaptation options to address climate change. Thus, the total costs of climate change can be reduced eventually, although part of the expense must be devoted to adaptation strategies. Finally, the aggregated costs obtained in this study for the 1.5 °C case are ${1.4}_{-0.4}^{+0.1}$ times costlier than the 2 °C case. This factor is lower than the factor of 2.2–3.7 reported in Rogelj et al. (2015) for achieving the 1.5 °C-consistent scenario and the medium 2 °C scenario; this difference is likely due to the assumption of moderate future emissions (i.e., the use of the SSP2) and the treatment of adaptation measures. In addition, the GDP losses also show narrower ranges with relatively lower levels compared to the corresponding idealized scenarios implemented for AR5; in Fig. 6.21 in IPCC (2014a), the 430–480 ppm CO_2_-eq scenarios are associated with GDP losses of approximately 3–9% in 2100,, whereas the 480–530 ppm CO_2_-eq scenarios are associated with GDP losses of approximately 4–10% in 2100. Again, the moderate future emissions and the introduction of adaptation may reduce the GDP losses in our study, whereas considering both socioeconomic and climate-related uncertainties may widen the range of uncertainty for the AR5 scenarios.

## Conclusions

This study evaluates emission pathways that can be followed to achieve the Paris 2 and 1.5 °C targets by 2100 while considering uncertainties in the carbon cycle and the climate system and explores how such uncertainties influence socioeconomic outcomes. Our results generally illustrate the significance of climate-related uncertainties in socioeconomic assessments of climate policies. We obtain the following specific findings.

First, the climate-related uncertainties lead to a difference of 20.5 GtCO_2_ in the 2100 CO_2_ emission levels corresponding to the upper and lower ends of the likely range for the 2 °C target; this difference is 12.0 GtCO_2_ for the 1.5 °C target. The total forcing in 2100 is estimated to be ${3.3}_{-0.6}^{+0.6}$ Wm^−2^ for the 2 °C target and ${2.5}_{-0.5}^{+0.4}$ Wm^−2^ for the 1.5 °C target.

Second, the climate change costs are significantly affected by the climate-related uncertainties. To achieve the 2 °C target, the carbon price in 2100 is ${482}_{-301}^{+250}$ USD(2005)/tCO_2_, whereas it is ${713}_{-215}^{+301}$ USD(2005)/tCO_2_ for the 1.5 °C target. The GDP losses in 2100 are estimated to be ${1.9}_{-0.7}^{+0.6}$% of the total gross output for the 2 °C target and ${2.0}_{-0.5}^{+0.7}$% for the 1.5 °C target.

## Electronic supplementary material

Below is the link to the electronic supplementary material.

Supplementary material 1 (DOCX 205 KB)
