# Supplementary material for: How do climate-related uncertainties influence 2 and 1.5 °C pathways?
Source: Sustain Sci. 2018 Jan 9;13(2):291–9. doi: 10.1007/s11625-017-0525-2 (PMC6086293; doi:10.1007/s11625-017-0525-2)
Supplement: Supplementary file 1 — Supplementary material 1 (DOCX 205 KB) [file 11625_2017_525_MOESM1_ESM.docx]

Supporting Information for “How do climate-related uncertainties influence 2 °C and 1.5 °C pathways?”

Xuanming SU^1^, Hideo SHIOGAMA^2^, Katsumasa TANAKA^2^, Shinichiro FUJIMORI^1,3^, Tomoko HASEGAWA^1,4^, Yasuaki HIJIOKA^1^, Kiyoshi TAKAHASHI^1^ and Jingyu LIU^1^

1. *Center for Social and Environmental Systems Research, National Institute for Environmental Studies (NIES), Tsukuba, Japan*
2. *Center for Global Environmental Research, National Institute for Environmental Studies (NIES), Tsukuba, Japan*
3. *Energy Program, International Institute for Applied Systems Analysis (IIASA), Laxenburg A-2361, Austria*
4. *Ecosystem Services and Management Program, International Institute for Applied Systems Analysis (IIASA), Laxenburg A-2361, Austria*

Estimating parameters for use in the simple climate model

Carbon cycle parameters adopted from MAGICC 6.0

For the carbon cycle parameters that are consistent with MAGICC 6.0, we extract them directly from the definitions used in MAGICC 6.0 (Meinshausen 2011a & 2011b), as shown in Table S1.

CO_2_ fertilization parameters

We use both the logarithmic and rectangular hyperbolic forms to simulate the CO_2_ fertilization effects, based on the approach used in MAGICC 6.0. Here, the logarithmic description is defined as

(1)

where *β* is the CO_2_ fertilization factor, $C_{{CO}_{2}}(t)$ is the atmospheric CO_2_ concentration in year t, $C_{{CO}_{2}}^{0}$ denotes the pre-industrial CO_2_ concentration (278 ppm), and *β_log_* is the fertilization coefficient.

The rectangular hyperbolic description is given by Eq. (2-4):

(2)

(3)

(4)

where *β_sig(t)_* is the effective CO_2_ fertilization factor at time t, and *C_b_* denotes the concentration at which the NPP is zero, which is taken to be 31 ppm [*_Gifford_*, 1993].

Therefore, the CO_2_ fertilization coefficient (*β_fert_*) is given by

(5)

where *β_m_* is the allocation coefficient between the two descriptions of the CO_2_ fertilization effects; its value is taken to be 1.100486, following MAGICC 6.0 [*_Meinshausen et al._* 2011a & 2011b].

Using all 4 RCPs [*Vuuren et al.* 2011; *Thomson et al.* 2011; *Masui et al.* 2011; *Riahi et al.* 2011] and their extensions reported in *Meinshausen et al.* [2011c], we re-estimated *β* and *β_m_* by minimizing

$\sum_{1765}^{2100} \left( \beta_{fert}\left( t \right)-\beta_{fert}^{mgc6}(t) \right)^{2}$ (6)

where $\beta_{fert}^{mgc6}(t)$ is the CO_2_ fertilization coefficient obtained from the output of MAGICC 6.0. The average results for the 4 RCPs are shown in Table S2, and the CO_2_ concentrations are shown in Figure S1.

Regrowth parameters

In contrast to MAGICC 6.0, the regrowth ($G_{i}\left( t \right), i\in[P, H,S]$_;_ P, H and S stand for the plant pool, the detritus pool and the soil pool, respectively) is here defined to be linearly related to a relaxation time ($\tau_{i}(t)$), where *a_i_* and *b_i_* are parameters that are estimated based on the outputs of MAGICC 6.0 using the 4 RCPs.

(7)

(8)

(9)

We estimate *a_i_* and *b_i_* by minimizing Eq. (10-12), which are shown below.

$\sum_{1765}^{2100} \left( G_{P}\left( t \right)-G_{P}^{mgc6}(t) \right)^{2}$ (10)

$\sum_{1765}^{2100} \left( G_{H}\left( t \right)-G_{H}^{mgc6}(t) \right)^{2}$ (11)

$\sum_{1765}^{2100} \left( G_{S}\left( t \right)-G_{S}^{mgc6}(t) \right)^{2}$ (12)

where $G_{P}^{mgc6}(t)$, $G_{H}^{mgc6}(t)$ and $G_{S}^{mgc6}(t)$ are outputs from MAGICC 6.0. Here, we estimate the regrowth parameters for each RCP scenario separately and average the values to produce the final estimates. The results can be found in Table S3.

Climate parameters

A two-box module is constructed, following DICE-2013R. We estimate the GMT above the preindustrial level, *T_AT_ (t)*, as

$T_{AT}\left( t \right)=T_{AT}\left( t-1 \right)+\xi_{1}\{f^{e}\left( t \right)-\xi_{2}T_{AT}\left( t-1 \right)-\xi_{3}[T_{AT}\left( t-1 \right)-T_{LO}\left( t-1 \right)]\}$ (13)

We also estimate the temperature increase in the deep ocean, *T_LO_ (t)*, by

$T_{LO}\left( t \right)=T_{LO}\left( t-1 \right)+\xi_{4}(T_{AT}\left( t-1 \right)-T_{LO}\left( t-1 \right))$ (14)

where $f^{e}\left( t \right)$ is the total effective radiative forcing. The parameter $\xi_{2}$ can be calculated directly using the assumptions regarding the climate sensitivity made in MAGICC 6.0 (*t_2xco2_*) and the forcing coefficient of doubling the CO_2_ concentration (*f_co22x_*) (Su *et al.* 2017), and the parameters $\xi_{1}$, $\xi_{3}$ and $\xi_{4}$ can be estimated by minimizing

$\sum_{1765}^{2100} \left( T_{AT}\left( t \right)-T_{AT}^{mgc6}(t) \right)^{2}$ (15)

where $T_{AT}^{mgc6}(t)$ represents output from MAGICC 6.0. Similarly, we estimate the climate parameters for each RCP scenario separately and average the values to produce the final estimates that can be found in Table S4. The temperature increases above the pre-industrial level are shown in Figure S2.

Table S1: Carbon cycle parameters adopted from the model emulation exercise using MAGICC 6.0 (Meinshausen 2011a & 2011b)

| No. | Parameter in SCM4OPT^*^ | Parameter in MAGICC 6.0 | BERN | CCSM1 | CLIMBER | FRCGC | HADLEY | IPSAL | LLNL | MPI | UMD2 | UVIC |
| --- | --- | --- | --- | --- | --- | --- | --- | --- | --- | --- | --- | --- |
| 1 | *σ_NPP_* | CO2_FEEDBACKFACTOR_GPP^**^ | 0.0107 | 0.0088 | 0.0380 | 0.0244 | -0.0032 | -0.0552 | -0.0004 | -0.0302 | -0.0307 | -0.0217 |
| 2 | *σ_rsp_* | CO2_FEEDBACKFACTOR_RESPIRATION | 0.0685 | -0.0161 | 0.0984 | -0.2000 | 0.1923 | -0.2000 | -0.0079 | -0.0242 | 0.0182 | 0.0057 |
| 3 | *σ_H_* | CO2_FEEDBACKFACTOR_DETRITUS | -0.1358 | -0.2798 | -0.2632 | 0.3995 | -0.0308 | -0.0958 | -0.2223 | -0.0576 | -0.3000 | -0.1809 |
| 4 | *σ_S_* | CO2_FEEDBACKFACTOR_SOIL | 0.1541 | 0.1522 | 0.2300 | 0.2236 | 0.0386 | 0.0912 | 0.2183 | 0.0680 | 0.1973 | 0.1056 |
| 5 | *ρ_ocn_* | OCEANCC_SCALE_IMPULSERESPONSE | 0.9493 | 1.6773 | 2.8733 | 1.1199 | 2.7146 | 0.0100 | 1.3745 | 1.2261 | 0.5673 | 1.7369 |
| 6 | *κ* | OCEANCC_SCALE_GASXCHANGE | 0.2040 | 0.1653 | 0.1610 | 0.2222 | 0.0532 | 0.0716 | 0.1606 | 0.2307 | 0.1606 | 0.2072 |
| 7 | *α_T_* | OCEANCC_TEMPFEEDBACK | 0.0846 | 0.1172 | 0.1162 | 0.0609 | 0.1386 | -1.2489 | 0.0933 | 0.0692 | -0.0429 | 0.1012 |
| 8 | *γ_ocn_* | OCEANCC_CONVLTN2INT_YRSAGO | 10.0000 | 10.0000 | 10.0000 | 10.0000 | 10.0000 | 10.0000 | 10.0000 | 10.0000 | 10.0000 | 10.0000 |
| 9 | *P_0_* | CO2_PLANTPOOL_INITIAL | 884.8584 | 870.4989 | 919.3811 | 484.2099 | 494.5423 | 547.8515 | 735.3877 | 351.4974 | 493.4998 | 621.1054 |
| 10 | *H_0_* | CO2_DETRITUSPOOL_INITIAL | 92.7738 | 56.7203 | 94.5397 | 32.6689 | 61.2050 | 66.4868 | 103.1582 | 72.1718 | 70.4359 | 52.2373 |
| 11 | *S_0_* | CO2_SOILPOOL_INITIAL | 1681.5250 | 1028.0560 | 1713.5320 | 592.1234 | 1109.3410 | 1205.0740 | 1869.7420 | 1308.1130 | 1276.6500 | 946.8014 |
| 12 | *F^0^_NPP_* | CO2_GPP_INITIAL | 66.2716 | 66.5843 | 64.4433 | 48.1558 | 61.0000 | 57.4992 | 66.8000 | 53.2807 | 53.0013 | 61.8993 |
| 13 | *F^0^_rsp_* | CO2_RESPIRATION_INITIAL | 12.2603 | 12.3181 | 11.9220 | 8.9088 | 11.2850 | 10.6374 | 12.3580 | 9.8569 | 9.8052 | 11.4514 |
| 14 | *Ρ_p2d_* | CO2_FRACTION_PLANT_2_DETRITUS | 0.9989 | 0.9990 | 0.9825 | 0.9990 | 0.3806 | 0.9990 | 0.9990 | 0.9989 | 0.9990 | 0.9990 |
| 15 | *δ_d2s_* | CO2_FRACTION_DETRITUS_2_SOIL | 0.0010 | 0.0010 | 0.0010 | 0.0618 | 0.0010 | 0.0010 | 0.0010 | 0.0010 | 0.0010 | 0.0010 |
| 16 | *ν_P_* | CO2_FRACTION_GPP_2_PLANT | 0.4483 | 0.4848 | 0.4517 | 0.6597 | 1.0000 | 0.8654 | 0.5740 | 0.2183 | 0.8853 | 0.6914 |
| 17 | *ν_H_* | CO2_FRACTION_GPP_2_DETRITUS | 0.3998 | 0.3472 | 0.4069 | 0.2265 | 0.0000 | 0.0000 | 0.3098 | 0.5732 | 0.0000 | 0.0000 |
| 18 | *δ_P_* | CO2_FRACTION_DEFOREST_PLANT | 0.7000 | 0.7000 | 0.7000 | 0.7000 | 0.7000 | 0.7000 | 0.7000 | 0.7000 | 0.7000 | 0.7000 |
| 19 | *δ_H_* | CO2_FRACTION_DEFOREST_DETRITUS | 0.0500 | 0.0500 | 0.0500 | 0.0500 | 0.0500 | 0.0500 | 0.0500 | 0.0500 | 0.0500 | 0.0500 |
| 20 | *C_b_* | CO2_GIFFORD_CONC_FOR_ZERONPP | 80.0000 | 80.0000 | 80.0000 | 80.0000 | 80.0000 | 80.0000 | 80.0000 | 80.0000 | 80.0000 | 80.0000 |
| 21 | *ψ* | CO2_NORGRWTH_FRAC_DEFO | 0.5000 | 0.5000 | 0.5000 | 0.5000 | 0.5000 | 0.5000 | 0.5000 | 0.5000 | 0.5000 | 0.5000 |
| 22 | *ρ_fert_* | CO2_FERTILIZATION_SCALE | 1.0413 | 1.0288 | 1.0275 | 1.0173 | 1.0254 | 1.0414 | 1.0666 | 1.0678 | 1.0137 | 1.0286 |

* See Eq. (21-47) in Su *et al.* (2017) for definitions.

** Only the remaining part of GPP, namely the NPP, is simulated in MAGICC 6.0.

Table S2: Parameters for fertilization factors used in this study, compared to MAGICC 6.0 (Meinshausen 2011a & 2011b)

| Model | No. | Parameter in SCM4OPT | Parameter in MAGICC 6.0 | BERN | CCSM1 | CLIMBER | FRCGC | HADLEY | IPSAL | LLNL | MPI | UMD2 | UVIC |
| --- | --- | --- | --- | --- | --- | --- | --- | --- | --- | --- | --- | --- | --- |
| SCM4OPT | 23 | *β* | CO2_FERTILIZATION_FACTOR | 0.67 | 0.46 | 0.54 | 0.27 | 0.59 | 0.81 | 1.10 | 1.13 | 0.17 | 0.59 |
|  | 24 | *β_m_* | CO2_FERTILIZATION_METHOD | 1.44 | 2.00 | 2.00 | 2.00 | 2.00 | 1.25 | 1.00 | 1.01 | 1.63 | 2.00 |
| MAGICC 6.0 | 23 | *β* | CO2_FERTILIZATION_FACTOR | 0.65 | 0.44 | 0.52 | 0.26 | 0.57 | 0.75 | 1.03 | 1.05 | 0.17 | 0.57 |
|  | 24 | *β_m_* | CO2_FERTILIZATION_METHOD | 1.10 | 1.81 | 1.95 | 2.00 | 2.00 | 2.00 | 1.00 | 1.00 | 1.44 | 1.95 |

Table S3: Regrowth parameters for the terrestrial carbon cycle

| No. | Parameter in SCM4OPT | BERN | CCSM1 | CLIMBER | FRCGC | HADLEY | IPSAL | LLNL | MPI | UMD2 | UVIC |
| --- | --- | --- | --- | --- | --- | --- | --- | --- | --- | --- | --- |
| 25 | *a_P_* | 19.7771 | 22.9832 | 19.3980 | 28.7760 | 60.7883 | 47.2993 | 29.9792 | 2.3368 | 45.5161 | 37.4072 |
| 26 | *b_P_* | -0.3905 | -0.5279 | -0.3632 | -1.3647 | -6.1372 | -3.3897 | -1.0616 | -0.0119 | -3.4380 | -1.8931 |
| 27 | *a_H_* | -0.4158 | -0.2964 | 1.1276 | -15.7508 | -25.0270 | -57.5299 | -27.8943 | 15.1559 | -67.8580 | -25.6812 |
| 28 | *b_H_* | 0.1988 | 0.2248 | -0.5132 | 16.3593 | 7.7586 | 33.8908 | 12.6311 | -6.7886 | 35.8033 | 15.4324 |
| 29 | *a_S_* | 10.0668 | 11.3337 | 7.7320 | 5.2590 | -206.1458 | 7.6043 | 7.6311 | 11.2316 | 5.8719 | 19.2061 |
| 30 | *b_S_* | -0.0607 | -0.1240 | -0.0427 | -0.0674 | 5.7298 | -0.0494 | -0.0320 | -0.0957 | -0.0283 | -0.3889 |

Table S4: Climate parameters adopted from the model emulation exercise using MAGICC 6.0 (indicated by ^*^) (Meinshausen 2011a & 2011b) and the tuned parameters used in the climate equations

| No. | 31 | 32 | 33 | 34 | 35 |
| --- | --- | --- | --- | --- | --- |
| Parameter in SCM4OPT | *t_2xco2_* | *f_co22x_* | *ξ_1_* | *ξ_3_* | *ξ_4_* |
| Parameter in MAGICC 6.0 | CORE_CLIMATESENSITIVITY^*^ | CORE_DELQ2XCO2^*^ | - | - | - |
| CCCMA_CGCM3_1_T47 | 2.9747 | 3.32 | 0.0692 | 0.3905 | 0.0034 |
| CNRM_CM3 | 2.9828 | 3.48 | 0.0692 | 0.3993 | 0.0024 |
| CSIRO_MK3_0 | 2.2352 | 3.47 | 0.0413 | 0.4639 | 0.0013 |
| GFDL_CM2_0 | 2.3059 | 3.50 | 0.0578 | 0.9000 | 0.0491 |
| GFDL_CM2_1 | 2.2820 | 3.50 | 0.0383 | 0.1867 | 0.0047 |
| GISS_MODEL_E_H | 2.5368 | 4.06 | 0.0692 | 0.4134 | 0.0014 |
| GISS_MODEL_E_R | 2.2569 | 4.06 | 0.0309 | 0.8677 | 0.0529 |
| IAP_FGOALS1_0_G | 2.4223 | 3.71 | 0.0526 | 0.8170 | 0.2285 |
| INMCM3_0 | 2.3515 | 3.71 | 0.0734 | 0.3307 | 0.0028 |
| IPSL_CM4 | 4.1475 | 3.48 | 0.0512 | 0.5111 | 0.0032 |
| MEDIUM_CMIP3_ECS3 | 3.0000 | 3.71 | 0.0535 | 0.3993 | 0.0022 |
| MIROC3_2_HIRES | 5.7344 | 3.14 | 0.0480 | 0.3324 | 0.0031 |
| MIROC3_2_MEDRES | 3.9961 | 3.09 | 0.0424 | 0.3955 | 0.0018 |
| MIUB_ECHO_G | 2.6256 | 3.71 | 0.0795 | 0.3054 | 0.0029 |
| MPI_ECHAM5 | 3.2292 | 4.01 | 0.0238 | 0.0000 | 0.7764 |
| MRI_CGCM2_3_2A | 2.4761 | 3.47 | 0.0625 | 0.3552 | 0.0021 |
| NCAR_CCSM3_0 | 2.1365 | 3.95 | 0.0408 | 0.8549 | 0.0868 |
| NCAR_PCM1 | 1.8957 | 3.71 | 0.0817 | 0.4887 | 0.0031 |
| UKMO_HADCM3 | 3.2074 | 3.81 | 0.0608 | 0.4147 | 0.0035 |
| UKMO_HADGEM1 | 2.9983 | 3.78 | 0.1018 | 0.8720 | 0.0223 |


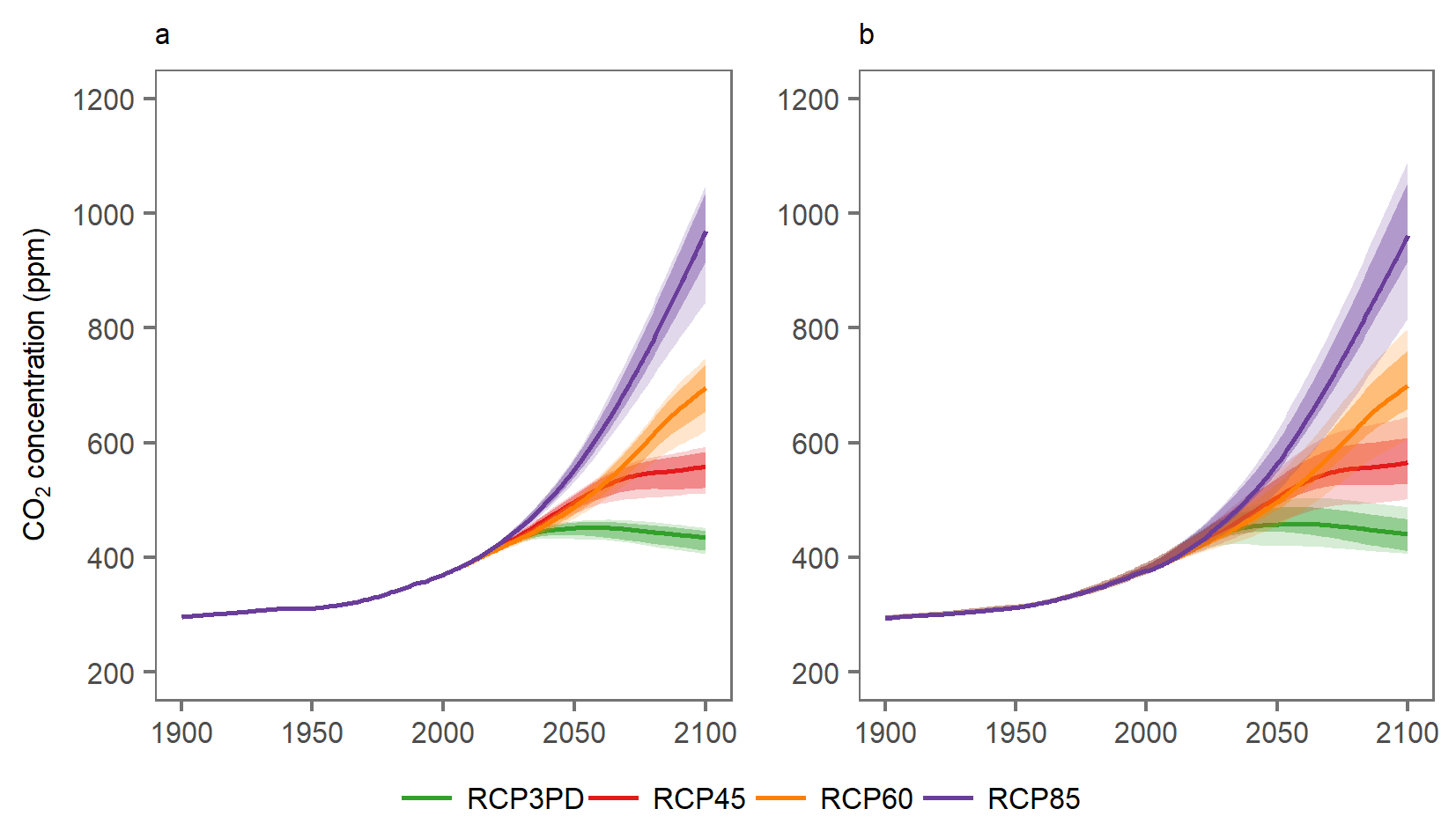


Figure S1: A comparison of the CO_2_ concentrations obtained using MAGICC 6.0 (a) and SCM4OPT (b) under the 4 RCPs. The outer ranges indicate the 17^th^ and 83^rd^ percentiles; the inner ranges indicate the 25^th^ and 75^th^ percentiles; and the solid-colored lines indicate the 50^th^ percentile.


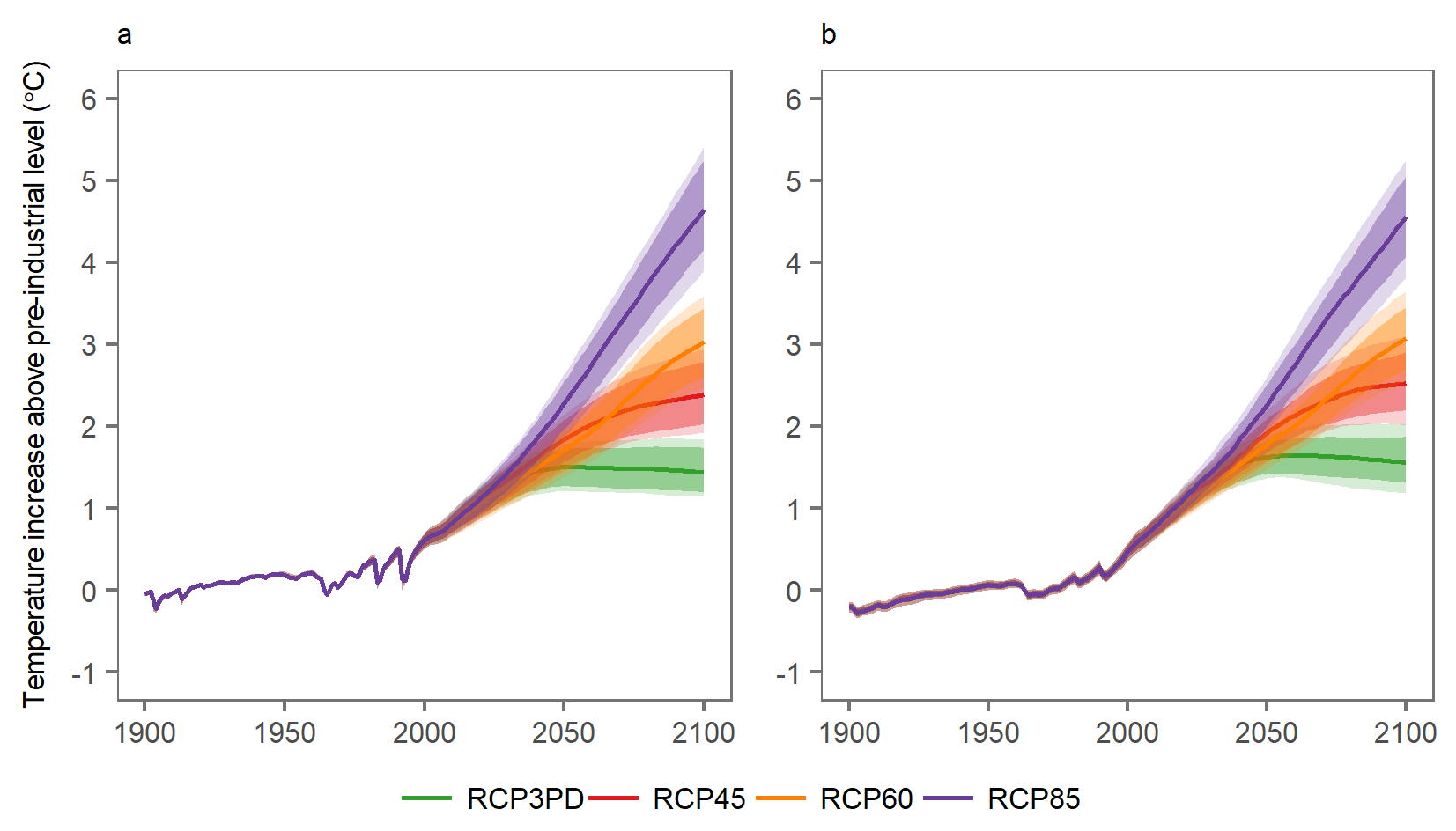


Figure S2: A comparison of temperature increases above the pre-industrial level between MAGICC 6.0 (a) and SCM4OPT (b) under the 4 RCPs. The outer ranges indicate the 17^th^ and 83^rd^ percentiles; the inner ranges indicate the 25^th^ and 75^th^ percentiles; and the solid-colored lines indicate the 50^th^ percentile.

Uncertainty pathways for CO_2_ emissions, GHG emissions, CO_2_ concentrations and temperature increases


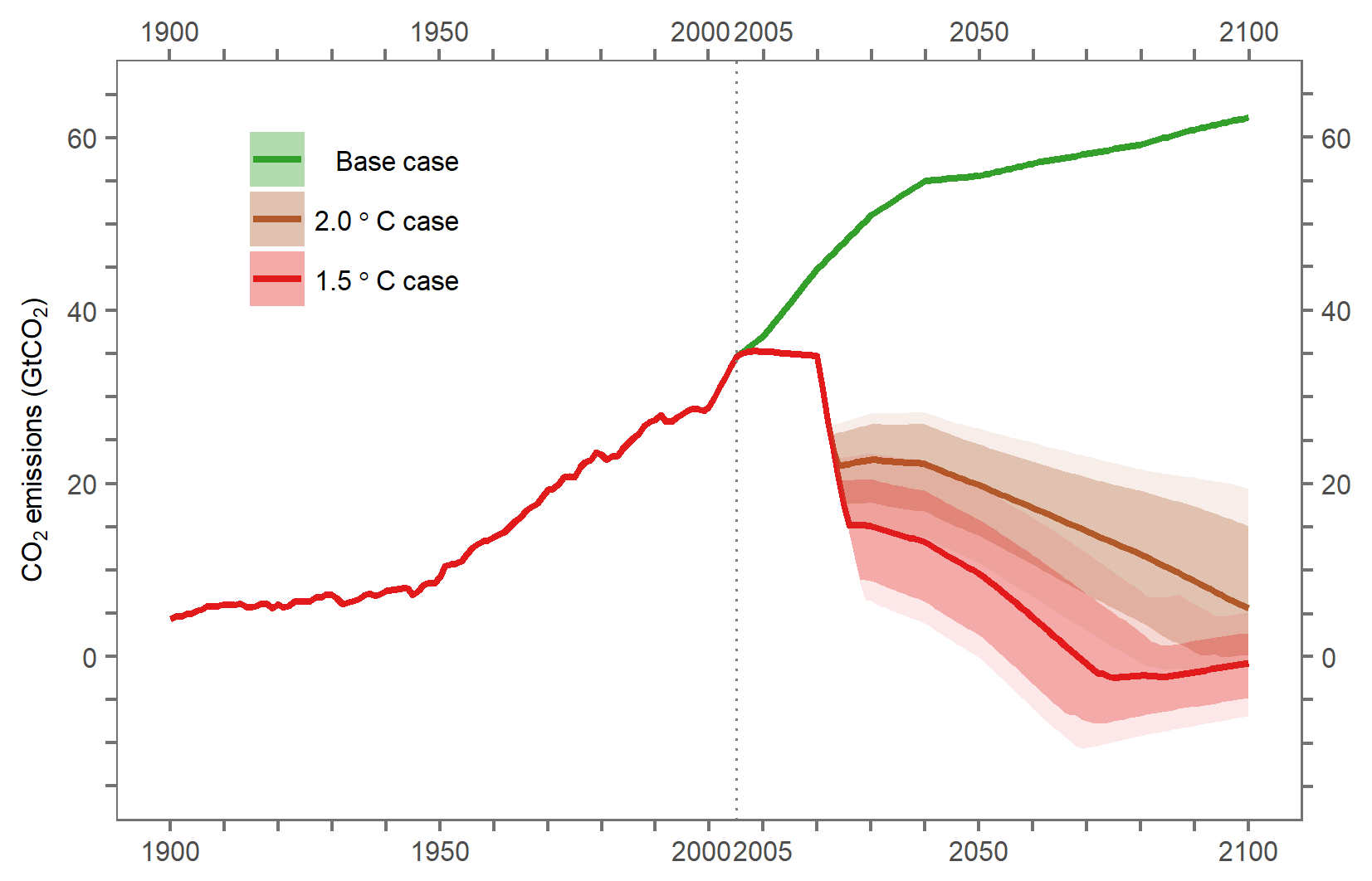


Figure S3: CO_2_ emissions from 1900 to 2100 for the base case, the 2 °C case and the 1.5 °C case. Historical data (1900-2004) are based on the historical emissions assumptions used in the RCPs (http://www.iiasa.ac.at/web-apps/tnt/RcpDb/).


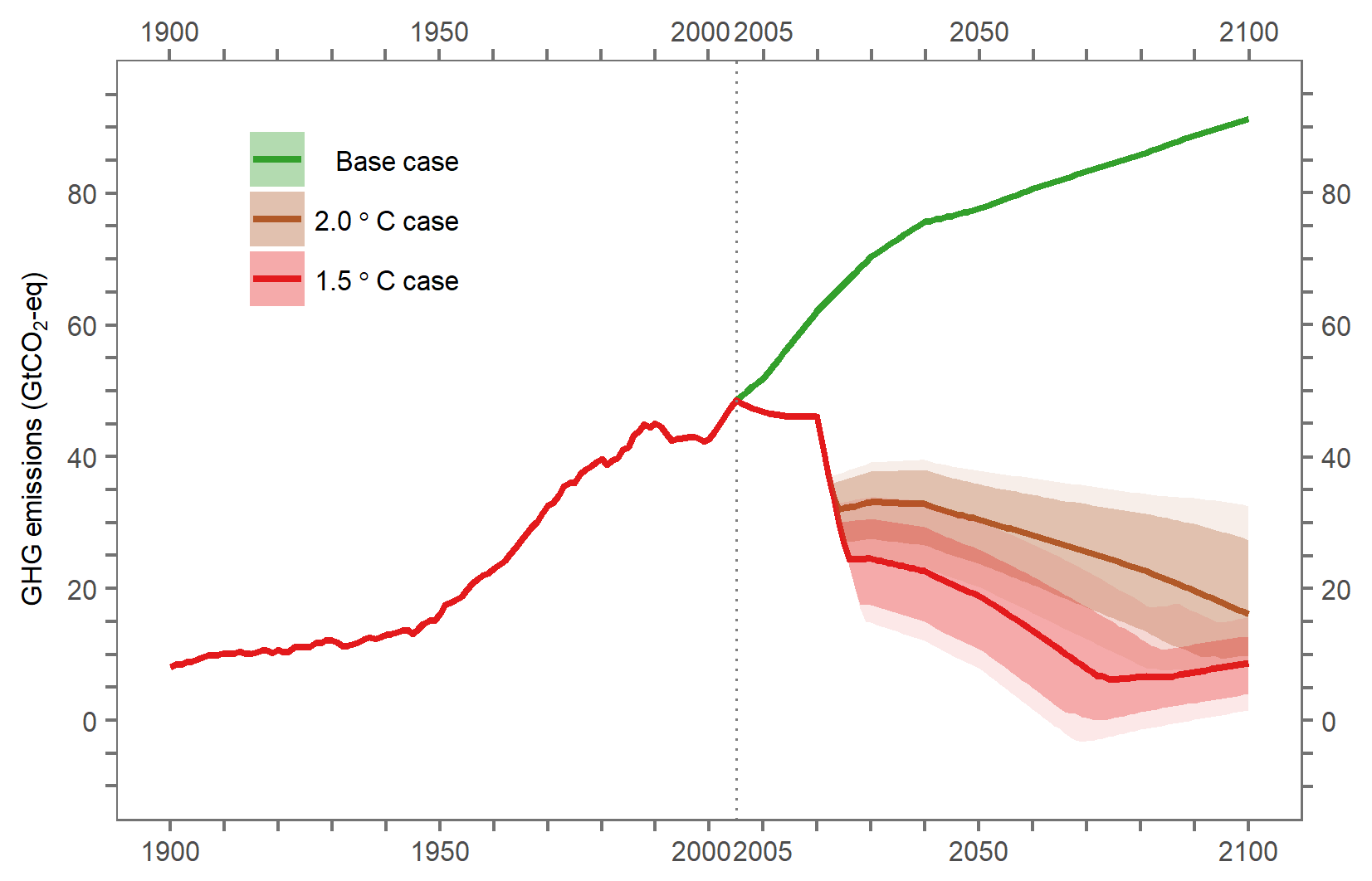


Figure S4: GHG emissions from 1900 to 2100 for the base case, the 2 °C case and the 1.5 °C case. Historical data (1900-2004) are based on the historical emissions assumptions used in the RCPs (<http://www.iiasa.ac.at/web-apps/tnt/RcpDb/>).


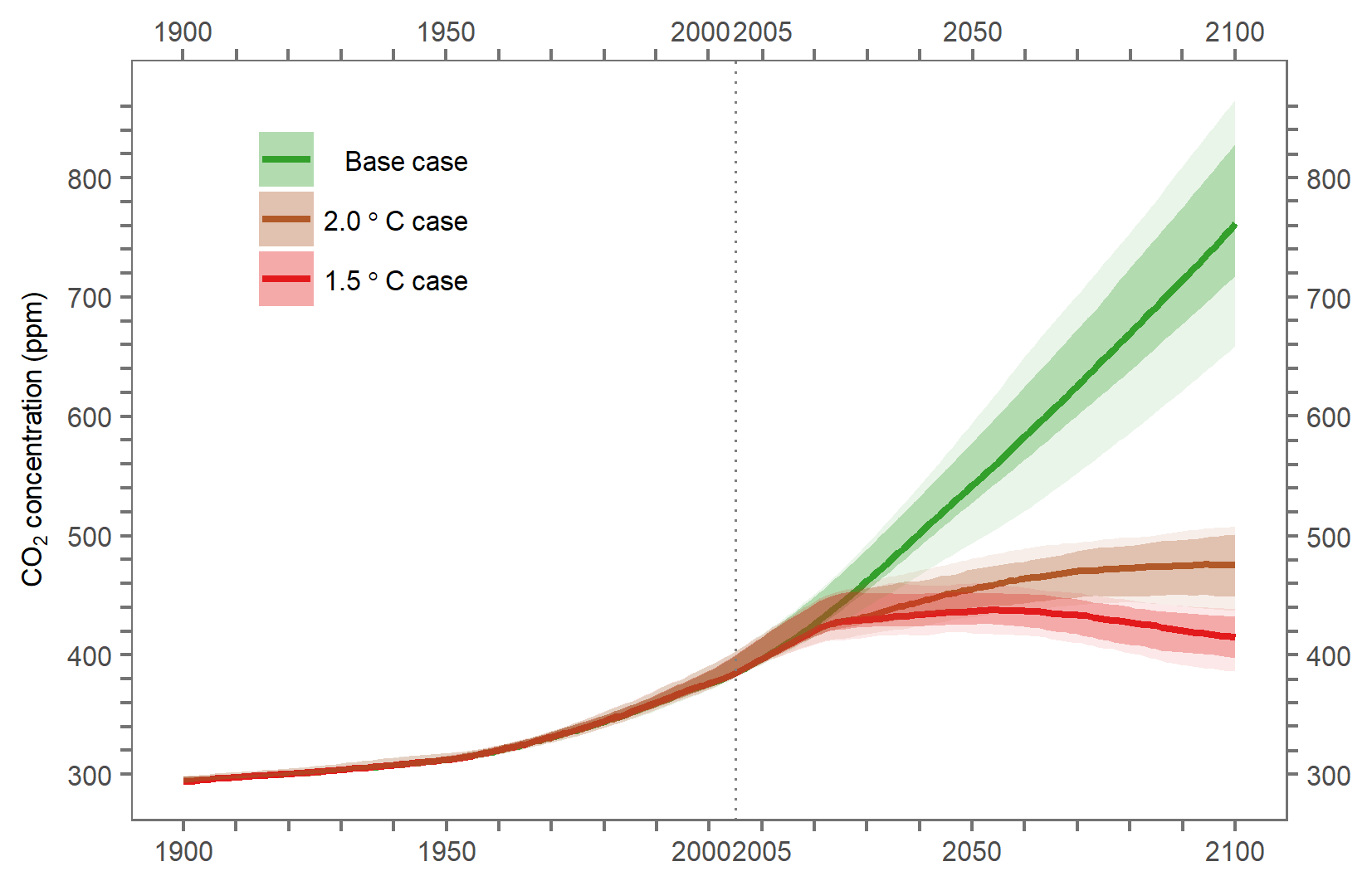


Figure S5: CO_2_ concentrations from 1900 to 2100 for the base case, the 2 °C case and the 1.5 °C case. Historical data (1900-2004) are calculated based on the historical emissions assumptions used in the RCPs (<http://www.iiasa.ac.at/web-apps/tnt/RcpDb/>).

**
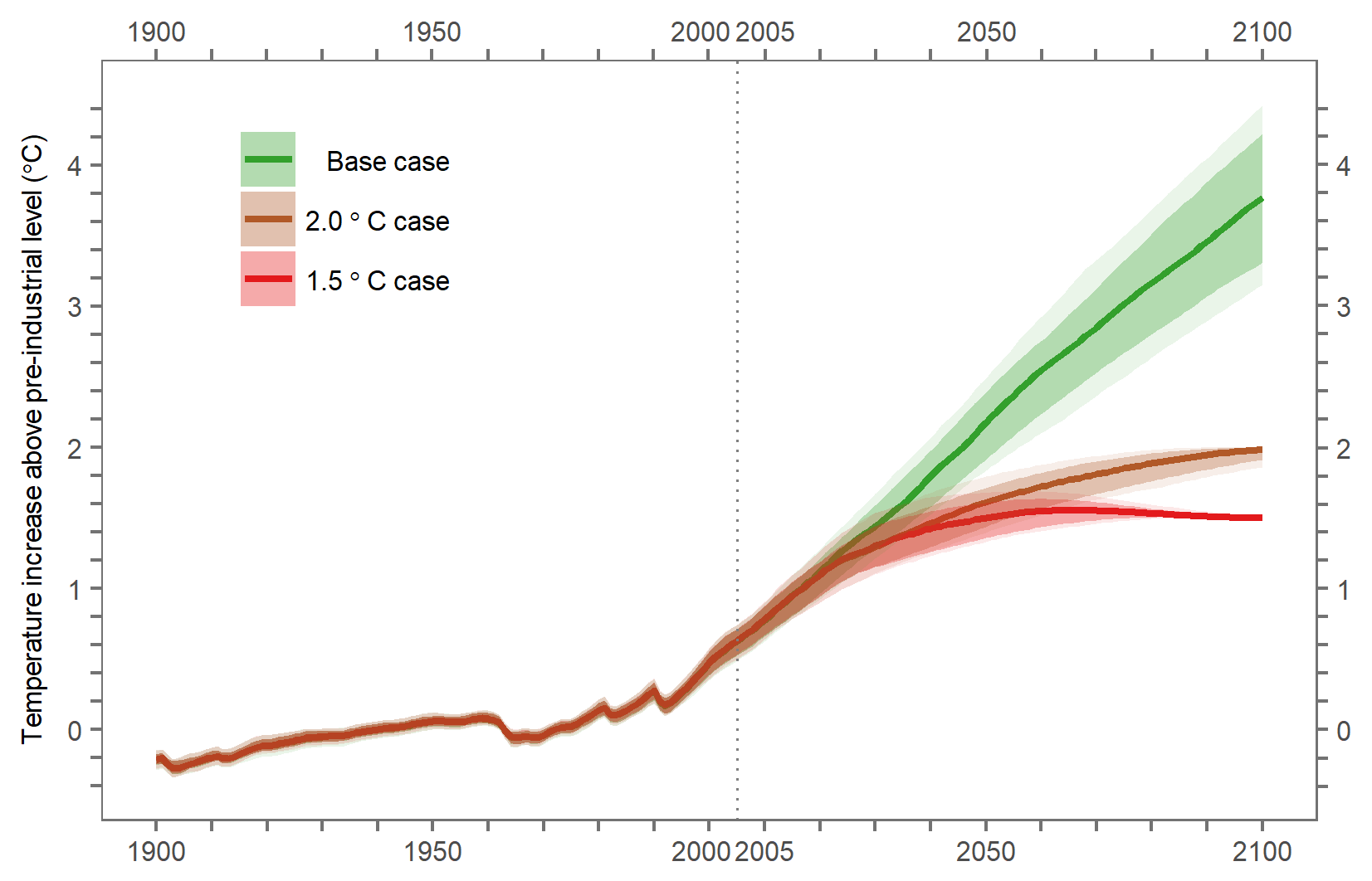
**

Figure S6: Temperature increase above the pre-industrial level from 1900 to 2100 for the base case, the 2 °C case and the 1.5 °C case. Historical data (1900-2004) are calculated based on the historical emissions assumptions used in the RCPs (<http://www.iiasa.ac.at/web-apps/tnt/RcpDb/>). The temperature increase is constrained to be below 2 °C or 1.5 °C after 2100 for the 2 °C and the 1.5 °C case, respectively.

**References**

Giffor RM (1993) Implications of CO_2_ Effects on Vegetation for the Global Carbon Budget. Springer Berlin Heidelberg, Berlin, Heidelberg, pp 159-199. DOI 10.1007/978-3642-84608-3_7

Masui T, Matsumoto K, Hijioka Y, Kinoshita T, Nozawa T, Ishiwatari S, Kato E, Shukla PR, Yamagata Y, Kainuma M (2011) An emission pathway for stabilization at 6 Wm^-2^ radiative forcing. Clim. Change 109(1-2): 59-76. DOI 10.1007/s10584-011-0150-5

Meinshausen M, Raper SCB, Wigley TML (2011a) Emulating coupled atmosphere-ocean and carbon cycle models with a simpler model, MAGICC6 - Part 1: Model description and calibration. Atmospheric Chemistry and Physics 11(4): 1417-1456. DOI 10.5194/acp-11-1417-2011

Meinshausen M, Wigley TML, Raper SCB (2011b) Emulating atmosphere-ocean and carbon cycle models with a simpler model, MAGICC6 - Part 2: Applications. Atmospheric Chemistry and Physics 11(4): 1457-1471. DOI 10.5194/acp-11-1457-2011

Meinshausen M, Smith SJ, Calvin K, Daniel JS, Kainuma MLT, Lamarque JF, Matsumoto K, Montzka SA, Raper SCB., Riahi K, Thomson A, Velders GJM, van Vuuren DP (2011c) The RCP greenhouse gas concentrations and their extensions from 1765 to 2300. Climatic Change 109(1): 213-241. DOI 10.1007/s10584-0110156-z

Riahi K, Rao S, Krey V, Cho C, Chirkov V, Fischer G, Kindermann G, Nakicenovic N, Rafaj P (2011) RCP 8.5 – A scenario of comparatively high greenhouse gas emissions. Clim. Change 109(1-2): 33-57. DOI 10.1007/s10584-011-0149-y

Su X, Takahashi K, Fujimori S, Hasegawa T, Tanaka K, Kato E, Shiogama H, Masui T, Emori S (2017) Emission pathways to achieve 2.0°C and 1.5°C climate targets. Earth's Future 5: 592-604. DOI 10.1002/2016EF000492

Thomson A, Calvin K, Smith S, Kyle GP, Volke A, Patel P, … Edmonds J (2011) RCP4.5: A pathway for stabilization of radiative forcing by 2100. Clim. Change 109(1-2): 77-94. [DOI 10.1007/s10584-011-0151-4](https://doi.org/10.1007/s10584-011-0151-4)

Vuuren D, Stehfest E, Elzen MJ, Kram T, Vliet J, Deetman S, … Ruijven B (2011) RCP2.6: Exploring the possibility to keep global mean temperature increase below 2°C. Clim. Change 109(1-2): 95-116. DOI 10.1007/s10584-011-0152-3
